# U.S. healthcare providers' knowledge, attitudes, beliefs, and perceptions concerning Chronic Fatigue Syndrome

**DOI:** 10.1186/1471-2296-11-28

**Published:** 2010-04-21

**Authors:** Dana J Brimmer, Frederick Fridinger, Jin-Mann S Lin, William C Reeves

**Affiliations:** 1 Division of Viral and Rickettsial Diseases, Centers for Disease Control and Prevention, Atlanta, GA, USA; 2 Division of Health Communication and Marketing, Centers for Disease Control and Prevention, Atlanta, GA, USA

## Abstract

**Background:**

Chronic fatigue syndrome (CFS) is a debilitating illness with particular difficulties for healthcare providers because there are no diagnostic signs or laboratory tests and because management aims to merely improve symptoms. Further complicating management, healthcare providers' awareness concerning CFS has not been rigorously assessed. The present study aimed to ascertain United States (U.S.) healthcare providers' awareness of CFS and to assess their knowledge, attitudes, and beliefs (KAB) related to diagnosis and management of the illness. This information forms the foundation for developing CFS educational strategies.

**Methods:**

We combined convenience and probability samples to measure CFS KAB among healthcare providers. In the convenience sample, 1,255 healthcare providers (81% response rate) from 13 professional conferences completed a 12-item form. Descriptive statistics were reported for 9 KAB item responses and chi-square tests were performed for examining their association with giving a diagnosis of CFS. We used principal component analysis to construct multidimensional subscales and perform a general linear model to examine factors associated with subscales. The probability sample involved data on 15 CFS-specific questions from 2006 and 2007 *DocStyles *web-based panel surveys collected from 2,750 physicians (average response rate 55%). We calculated descriptive and chi-square statistics. The significance was set at two-tailed with the alpha level of 0.05.

**Results:**

Healthcare providers in both samples were aware of CFS and exhibited a high level of knowledge. Overall, 96% of respondents in the *DocStyles *(probability) sample had heard about CFS. Healthcare providers in the conference (convenience) sample demonstrated good KAB scores; physicians' scores were highest on KAB scales and lowest in perception. Nurses' scores were lowest in knowledge. More than 40% of physicians reported ever giving a CFS diagnosis and in the *DocStyles *(probability) sample more than 80% of physicians correctly identified CFS symptoms. Physicians reported professional journals, the Internet, and continuing education programs as the top 3 sources from which they obtain CFS information.

**Conclusions:**

Findings from these combined samples fill a gap in the evidence-base of U.S. healthcare providers' and knowledge, attitudes, and beliefs concerning CFS. Importantly, respondents in both samples expressed similar knowledge, attitudes, beliefs and perceptions. Awareness was high and negative attitudes were low. The primary areas for future education should address diagnosis and management of CFS and should be delivered through those venues providers indicated they primarily use. Data from this study provide a benchmark for evaluation the success of these future efforts.

## Background

Chronic fatigue syndrome (CFS) is a debilitating illness [1] that presents unique difficulties for healthcare providers because there are no diagnostic signs, laboratory abnormalities or biomarkers and management aims to improve symptoms; no definitive therapy has been identified. Adding to this, the symptoms comprising CFS are similar to those of many medical illnesses, which must be ruled out [2,3]. Patients with CFS often require multiple office visits and insurance may not cover associated evaluations and lab tests. Further complicating diagnosis and management, some patients perceive skepticism or lack of awareness concerning CFS as a legitimate illness among providers (in particular physicians), which can lead to mistrust and a challenge to patients identity [4,5]. Finally, psychiatric comorbidities (e.g., depression) are common among CFS patients [6] and this poses additional challenges for healthcare providers [3,7].

These problems may in large part explain why only 50% of individuals with CFS have consulted a physician for their illness and fewer than 20% of people with CFS have been diagnosed and treated for the illness [8,9]. Educating providers concerning CFS should improve identification, diagnosis, and management of CFS, but educational activities must be based on providers' current knowledge, attitudes, and beliefs in order to target major current weaknesses. Knowledge, attitudes, and beliefs (KABs) have been studied in the United Kingdom (U.K.), the Netherlands, and Australia, but research in the United States (U.S.) is limited. In the U.K., Raine et al report that primary care physicians feel uncertain or dismissive of CFS, and that these perceptions may pose a barrier to illness management [10]. A separate study reported that despite recent guidelines on CFS management in the U.K. and positive attitudes towards CFS, only 72% of physicians recognized CFS clinically [11]. Bowen et al also reported that 49% of physicians identified the clinical hallmarks of CFS, yet knowledge was not a factor in the self-efficacy of giving a diagnosis [11]. Thomas and Smith found 56% of general practitioners believed CFS as an illness and of this group, 67% reported giving a diagnosis of CFS [12].

In the Netherlands, Scheeres et al showed that physicians informed about CFS reported higher levels of knowledge and better attitudes towards the illness [13]. And, in the same study physicians receiving CFS education were significantly more likely to refer patients for cognitive behavioral therapy compared to physicians who did not receive education [13]. In Australia, 27% of practitioners reported that they did not believe CFS as a syndrome, and yet 71% of the sample stated they had treated a patient for CFS [14]. This study also found that practitioners, who were younger, female, and practiced in small settings, were significantly more likely to accept CFS as an illness. Steven and colleagues state that practitioners who acknowledged CFS as a distinct illness reported more accurate knowledge of CFS (e.g., diagnostic characteristics) and identified appropriate management strategies [14]. One study in the United States did examine attitudes towards treating CFS patients in medical students. However, the study population and small sample size inhibit generalizability to physician populations [15].

We examined KABs about CFS among physicians and other healthcare providers from two different populations in the United States. Our research had three objectives: 1) ascertain awareness of CFS among healthcare providers; 2) assess the knowledge, attitudes, and beliefs related to CFS diagnosis and treatment, and 3) determine where physicians obtain information regarding CFS.

## Methods

We used convenience and probability sampling methods separately on two different populations regarding knowledge, attitudes, and beliefs concerning CFS by healthcare providers in the United States. In the first sample, we queried a convenience sample of healthcare providers from 13 national-level provider conferences around the country between February 2006 and October 2007. Conferences focused on physicians, nurse practitioners, physician assistants, nurses, or allied health providers and conference size ranged from 1,410 to 7,900 attendees. We staffed a CDC-sponsored CFS education booth in the conference exhibit hall, and for each conference an exhibit booth fee was paid per industry standard. For all conferences we advertised the CFS education booth through advertisements in conference programs, pre-event mailers, and partial conference sponsorships. The education booth displayed and provided free CFS materials such as journal articles, management guidelines, and reference bibliographies and also offered access to free CFS CME courses.

As part of the exhibit, a limited number (75-100 per conference) of USB sticks with CFS educational information were made available to conference participants on a "first-come, first-serve basis." The numbers of USB sticks per conference were decided a priori. In exchange for a USB stick, and to help evaluate the awareness booth activities, conference attendees were asked, but not required, to fill out an anonymous, one-page 12-item CFS KAB form. No identifying information was collected. Permission to dispense USB sticks and collect KAB information was granted by conference organizers and this follows a standard approach used by conference exhibitors.

Content validity of the KAB form was assessed from a pool of questions derived from a panel of experts in CFS research. It included 9 items preliminarily assessing CFS knowledge, attitudes, and beliefs, and 3 questions asking "Have you ever given a diagnosis of chronic fatigue syndrome" (yes, no, or not applicable); "Type of setting do you practice in" (hospital, private practice, group practice, academic, community, or other); and "Type of degree" (doctor of medicine (MD), doctor of osteopathic medicine (DO), nurse practitioner (NP), physician's assistant (PA), registered nurse (RN), occupational therapist (OT), doctor of philosophy (PhD), Masters degree, and other). Respondents could identify more than one practice setting and degree.

Descriptive statistics were reported for the 9 KAB item responses and chi-squared tests were performed to examine their association with giving a diagnosis of CFS. We also analyzed the KAB questions by using exploratory principal components analysis with a varimax rotation. Both a theoretical basis and Suhr's recommendations (variables on same factors share conceptual meanings and variables on different factors differ conceptually) guided variable selection and interpretation of factors [16]. We chose to keep items greater than 0.40 for each factor. The nine KAB questions were measured on scale ranging from 1 = strongly disagree and 7 = strongly agree. A general linear model examined the associations of KAB scales with the factors, CFS diagnosis, practice setting, and type of degree.

In the second sample, probability sampling method was employed through *DocStyles*, a survey conducted by Porter Novelli (a CDC contractor) in July and August 2006 and 2007. *DocStyles *is a web-based panel survey developed by Porter Novelli, with input from federal public agencies as well as other profit and non-profit organizations. The CDC submitted questions for the survey in 2006 and 2007. The panel sample stemmed from the Epocrates Honor Panel http://www.epocates.com, consisting of 142,000 verified physicians [17]. A sample is randomly selected from the master database to match the American Medical Association (AMA) files in terms of age, sex, and region, which has information on all physicians in the United States, both members and non-members [18]. This sample was screened to include United States physicians practicing in individual, group, or hospital practices; those in an active practice; and those practicing for the last three years. In 2006, the goal was to recruit 1000 primary care physicians and 250 pediatricians. In 2007, the sample was expanded to also include 250 obstetricians and gynecologists (OB/GYN). Participating physicians received an honorarium, $30 in 2006, and $45 to $55 in 2007, and were not required to participate in the survey and could opt out at anytime.

In 2006, the *DocStyles *survey contained 41 questions with multiple sub-parts, and in 2007, the survey had 69 questions with multiple sub-parts. There were fifteen CFS questions for both years of the survey. We measured knowledge by asking about CFS symptoms, and attitudes and beliefs by asking agreement to eight statements. Physicians were further asked if they had heard of CFS in the last six months, and if so, from what source the information originated, and if they had ever made a diagnosis of CFS. No personal identifiers were included in the survey and respondents could exit the web survey at anytime. Descriptive and frequency distributions, and chi-square statistics were reported in tables.

## Results

### Conference Healthcare Providers

Over a 21 month period, 4,065 healthcare providers at 13 conferences visited the CFS education booth. A total of 1,550 USB sticks were made available at all 13 conferences, and of these, 1,255 were requested for an 81% response rate. Characteristics of the sample are shown in Table 1. Physicians comprised about half (n = 585) of the sample, nurse practitioners or physician assistants accounted for 30% (n = 378), occupational therapists 10% (n = 124), and nurses 6% (n = 76). Most reported practicing in a hospital (26%) or private practice (27%).

**Table 1:** Characteristics of Healthcare Providers Conference sample (n = 1255).

	Frequency	Percent
**Degree**		
MD/DO	585	46.6
NP/PA	378	30.1
RN	76	6.1
OT	124	9.9
PhD/Masters	48	3.8
Other	38	3.0
Missing	6	0.5

**Practice Setting**		
Hospital	326	26
Private Practice	331	26.4
Group Practice	209	16.7
Academic	105	8.4
Community	98	7.8
Other	167	13.3
Missing	19	1.5

**Ever given a CFS diagnosis**		
Yes	512	40.8
No	615	49
Not Applicable	87	6.9
Missing	41	3.3

Forty-one percent (n = 512) of the sample reported ever given a diagnosis of CFS. Of these 71% (n = 366) were physicians, 23% (n = 116) nurse practitioners or physician assistants, 2% (n = 10) PhD/Masters, 3% nurses (n = 13), 1% (n = 6) occupational therapists, and other <1% (n = 1). The majority of physicians (35%) were affiliated with private practice; nurse practitioners and physician assistants were primarily affiliated with hospitals (28%); nurses with hospitals (60%); and occupational therapists with hospitals (33%). Practice setting was correlated with giving a diagnosis as 37% (n = 186) of those who gave a diagnosis were affiliated with a private practice, 21% (n = 106) with a hospital, 20% (n = 103) with a group practice, and 8% (n = 38) with an academic setting.

Responses to the 9 KAB questions were used in an exploratory principal components factor analysis and resulted in a four-factor best solution (eigenvalues greater than 1) that explained 73% of total variance (Table 2). Construct names were derived after reviewing content of the components from factor analysis: Attitude, Belief, Knowledge, and Perception. *Attitude *(20% of the variance) included three statements: 1) "CFS as not a big problem as media suggests" 2) "People with CFS are just depressed" 3) and, "If people with CFS rest they will get better." Scores for this factor ranged from 3 to 21, with a high score indicating more negative attitudes towards CFS. *Belief *(19% of the variance) included 2 items based on comparisons to other illnesses: 1) "CFS more difficult to treat and manage" 2) and, "CFS more difficult to diagnose." Scores for this factor ranged from 2 to 14, high scores indicating realistic beliefs about diagnosing and treating CFS. The *Knowledge *factor (18% of the variance) included 2 items: 1) "Criteria for a CFS diagnosis are 6 months or more of fatigue, debilitating fatigue, and 4 of the 8 symptom criteria" and 2) "CFS can be diagnosed using 3 different questionnaires: the Medical Outcomes Survey Short Form-36 (SF-36), Multidimensional Fatigue Inventory (MFI), and CDC Symptom Inventory." Scores ranged from 2 to 14, high scores indicating a good working knowledge of CFS. *Perception *comprised the last factor and accounted for 15% of the variance. *Perception *included: 1) "Majority of people with CFS have a high socio-economic status" and, 2) "Most people with CFS were competitive and driven before they got sick." Scores ranged from 2 to 14, with high scores indicating agreement of the statement. Reliability for each factor of *Attitude*, *Belief*, *Knowledge*, and *Perception*, as measured with standardized Cronbach's alpha were 0.66, 0.81, 0.73, and 0.56 respectively.

**Table 2:** Constructs Resulted from Factor Analysis on CFS Knowledge, Attitudes, and Belief in Conference Healthcare Providers (n = 1255)

Item	Rotated Factor Pattern^a^				Mean (SD)^d^
	Factor 1	Factor 2	Factor 3	Factor 4	
**Attitude**					
CFS is not as big a problem as the media suggests	***.772***	-.044	-.001	-.079	7.42 (3.38)
People with CFS are just depressed	***.771***	.050	-.117	.052	
If people with CFS rest then they will get better	***.769***	-.110	-.043	.148	

**Belief**					
Compared to other illnesses, CFS is more difficult to treat and manage	-.053	***.901***	.131	.066	10.19 (2.74)
Compared to other illnesses, CFS is more difficult to diagnose	-.036	***.896***	.129	.075	

**Knowledge**					
CFS can be diagnosed using the MFI^b^,	-.002	.068	***.888***	.133	10.05 (2.44)
SF-36^b^, and CDC Symptom Inventory^b^					
Criteria for a diagnosis of CFS	-.156	.221	***.824***	.124	

**Perception**					
Majority of people with CFS have a high	-.151	.155	-.003	***.831***	8.12 (2.55)
SES					
Majority of CFS people were competitive, driven to achieve, and compulsive before they got sick	-.054	-.017	.305	***.787***	

Variance explained by each factor^c^	20.41%	19.00%	17.88%	15.37%	

^a ^Factor method: principal components with varimax rotation

^b ^Multidimensional Fatigue Inventory, the Short Form 36, and the CDC Symptom Inventory

^c ^Total variance = 72.66

^d ^Scale: *Attitude *3 (strongly disagree) to 21 (strongly agree); *Belief *2 (strongly disagree) to 14 (strongly agree); *Knowledge *2 (strongly disagree) to 14 (strongly agree); *Perception *2 (strongly disagree) to 14 (strongly agree)

Data for the conference healthcare providers KABs are shown in Table 3. Except for the statement "Majority of people with CFS were more competitive, driven to achieve, and compulsive before getting sick," in which 43% of respondents agreed, most healthcare providers were generally positive towards CFS attitudes and beliefs, and showed good levels of knowledge. Respondents disagreed that CFS is not as big of a problem as the media suggests and that the majority of people with CFS are from a higher socio-economic strata. Healthcare providers agreed with statements that compared to other illnesses CFS is more difficult to diagnose (70%) and more difficult to treat and manage (72%).

**Table 3:** Conference Healthcare Providers Knowledge, Attitudes, and Beliefs of CFS (n = 1255).

	Agree^b^ n (%)	Disagree n (%)	Neutral n (%)	Mean^a^
CFS is not as big a problem as media suggests	128 (10)	951 (76)	162 (13)	2.48
People with CFS are just depressed	147 (12)	946 (75)	157 (13)	2.46
If people with CFS rest then they will get better	116 (9)	960 (77)	172 (14)	2.48
Compared to other illnesses CFS is more difficult to treat and manage	899 (72)	160 (13)	189 (15)	5.13
Compared to other illnesses CFS is more difficult to diagnose	881 (70)	194 (16)	177 (14)	5.06
CFS can be diagnosed using MFI, SF-36, and CDC Symptom Inventory^c^	615 (49)	159 (13)	443 (35)	4.69
Criteria for a CFS diagnosis are fatigue 6 months or longer, debilitating fatigue, and 4 of the 8 symptom criteria	948 (75)	113 (9)	184 (15)	5.36
Majority of people with CFS have a high SES	371 (30)	453 (36)	418 (33)	3.84
Majority of people with CFS were competitive, driven to achieve, and compulsive before getting sick	541 (43)	360 (28)	331 (26)	4.28

^a^Mean is for range of 1 - 7 (1 = strongly disagree to 7 = strongly agree)

^b^Agree = values 5, 6, 7; Disagree = values 1, 2, 3; Neutral = value 4

^c ^Multidimensional Fatigue Inventory (MFI), Medical Outcomes Survey Short Form-36 (SF-36),

We examined the impact of ever having made a CFS diagnosis on KABs (Table 4). A past history of giving a CFS diagnosis was significantly associated with the two Knowledge components, "Criteria for CFS" (p < 0.001) and "CFS can be diagnosed using MFI, SF-36, CDC Symptom Inventory" (p < 0.01); one Attitude statement "People with CFS are just depressed" (p < 0.05); and the Belief factor "Compared to other illness CFS more difficult to diagnose (p < 0.05) and treat and manage (p < 0.01).

**Table 4:** Conference Healthcare Providers History of CFS diagnosis and CFS KABs (n = 1255).

Have you ever given a diagnosis of CFS n (%)		Yes			No		
	Agree^a^	Disagree	Neutral	Agree	Disagree	Neutral	χ^2^
Criteria for CFS diagnosis: fatigue > 6 months and 4 of 8 symptom criteria	434 (85)	45 (9)	33 (6)	425 (70)	59 (10)	122 (20)	45.50***
CFS can be diagnosed using MFI, SF-36, and CDC Symptom Inventory^c^	284 (57)	58 (12)	158 (32)	278 (47)	85 (14)	231 (39)	10.87**
Majority of people with CFS were competitive, driven to achieve, compulsive before sick	227 (45)	140 (28)	134 (27)	262 (43)	174 (29)	168 (28)	0.41
CFS is not as big a problem as media suggests	56 (11)	392 (77)	60 (12)	59 (10)	459 (76)	90 (15)	2.14
Majority of people with CFS have a high SES	159 (31)	187 (37)	161 (32)	183 (30)	207 (34)	218 (36)	2.14
If people with CFS rest then they will get better	55 (11)	390 (76)	66 (13)	53 (9)	470 (77)	88 (14)	1.72
People with CFS are just depressed	70 (14)	386 (76)	54 (11)	59 (10)	464 (76)	89 (15)	7.45*
Compared to other illnesses CFS more difficult to diagnose	344 (67)	94 (18)	73 (14)	449 (73)	79 (13)	85 (14)	6.91*
Compared to other illnesses CFS more difficult to treat/manage	391 (77)	64 (13)	54 (11)	428 (70)	74 (12)	109 (18)	11.76**

* p < 0.05; ** p < 0.01; *** p < 0.001

^a^Agree = values 5, 6, 7; Disagree = values 1, 2, 3; Neutral = value 4

^c ^Multidimensional Fatigue Inventory (MFI), Medical Outcomes Survey Short Form-36 (SF-36),

#### The relationship of KAB and Perception to education of healthcare providers

Three scales, *Attitude, Belief, and Knowledge*, were associated with providers' education attainment (e.g. degree). Compared to nurses (mean = 8.1), nurse practitioners and physician assistants, occupational therapists, and the PhD/Masters group had lower *Attitude *scores. There was no difference in *Attitude *between physicians and nurses. Physicians, nurse practitioners and physician assistants, occupational therapists, and PhD/Masters had statistically significantly higher *Belief *scores than nurses (mean = 9.1). In terms of *Knowledge*, physician and nurse practitioners and physician assistants had higher scores than nurses (mean = 9.2; p < 0.05). Compared to nurses, there was no statistically significant difference in occupational therapists and the PhD/Masters group in terms of *Knowledge*. (See Additional file 1, Supplementary Table 1).

#### The relationship of KAB and Perception to practice setting of healthcare providers

Among the four scale scores, *Attitude *and *Perception *scores were associated with providers' practice settings. Compared to healthcare providers affiliated with the community, providers in hospitals had higher *Attitude *scores (mean = 7.8) than those working in community settings (p = 0.03) and providers in private practice also had higher *Attitude *scores than those working in community settings (mean = 6.9, p = 0.02). Among all types of practice settings, only healthcare providers in all other practice setting group had a significant lower *Perception *score than healthcare providers in communities (mean 7.6 vs. 8.3, p = 0.01). (See Additional file 1, Supplementary Table 1).

#### The relationship of KAB and Perception to CFS diagnosis

*Knowledge *and *Perception *scores were associated with having diagnosed patients as CFS. Providers who had diagnosed CFS had a higher *Knowledge *scores than those who had never diagnosed the illness (mean 10.4 vs. 9.8, p < 0.0001), but their *Perception *scores were similar (p = 0.89). Healthcare providers who were not applicable to give a CFS diagnosis had a lower *Perception *score than healthcare providers who never gave a CFS diagnosis before (mean 7.5 vs. 8.2, p = 0.03). (See Additional file 1, Supplementary Table 1).

After adjusting for education, practice setting, and CFS diagnosis, professional degree (e.g., MD, PA) remained significantly associated with *Knowledge*, *Attitude*, and *Belief*, but diagnosis did not. After the adjustment, practice setting remained only significantly associated with *Attitude*.

### DocStyles Physicians

The 2006 DocStyles study collected responses from 1,455 physicians (response rate 61%). However, the final sample size was 1,250 due to a random data storage error that occurred during an overnight backup procedure in which the programmer accidently overwrote the data. No particular demographics were affected as one days' worth of data (205 physicians) was lost but the original quotas were obtained. In 2007, 1,500 physicians completed surveys for a 48% response rate. Demographics for both study years are displayed in Table 5.

**Table 5:** Demographics of DocStyles Physicians.

	2006		2007		
	**PCP^a^** **n (%)**	**PED^b^** **n (%)**	**PCP** **n (%)**	**PED** **n (%)**	**OB/GYN^c^** **n (%)**
Sample size	1000 (80)	250 (20)	1000 (66.6)	250 (16.6)	250 (16.6)
Sex					
Male	720 (72)	127 (51)	690 (69)	167 (67)	100 (40)
Female	280 (28)	122 (49)	310 (31)	83 (33)	150 (60)
Age (average)	44.2	45.0	44.1	44.4	45.2
Years in Practice (average)	13.7	14.9	13.5	14.9	14.5
Region					
NE	240 (24)	40 (16)	240 (24)	71 (29)	67 (27)
North Central	220 (22)	75 (30)	250 (25)	52 (21)	57 (23)
South	320 (32)	80 (32)	300 (30)	75 (30)	85 (34)
West	220 (22)	52 (21)	210 (21)	52 (21)	42 (17)
Number of patients per week (average)	121.0	124.8	123.0	109.5	119.6
Number of physicians in group (average)	12.8	8.9	18.0	11.6	12.0
Have you ever diagnosed a patient with CFS?	521 (52)	50 (20)	471 (47)	41 (16)	24 (10)

^a ^Primary care physician (Family/General Practitioner, Internist)

^b ^Pediatrician

^c ^Obstetrician/Gynecologist

DocStyles physicians were asked, "Before today, have you ever heard of chronic fatigue syndrome (CFS)?" In 2006, 97% (n = 1212) responded yes as did 96% (n = 1441) in 2007. Table 6 shows the data in response to the 7 CFS opinion statements. When asked, "There is enough information available to clinicians to diagnosis CFS," 25% (n = 308) agreed in 2006 and 30% (n = 448), in 2007. In both surveys 20% of physicians agreed with the statement "I believe CFS is only in the patient's head." For the statement, "There are several treatment options available for people with CFS," 43% (n = 534) agreed with the statement in 2006 and 49% (n = 738) in 2007. Physicians were also queried about quality of life for both CFS patients and their caregivers or families. When asked, "CFS can impair a person's quality of life," 90% (n = 1353) in the 2006 sample, and 87% (n = 1088) in the 2007 sample agreed. As for the question, "CFS can impair the quality of life for the patient's family or caregiver," 86% (n = 1076) in 2006, and 89% (n = 1341) in 2007, agreed.

**Table 6:** DocStyles Physicians CFS Knowledge, Attitudes, and Beliefs.

		**2006^a^** **n (%)**			**2007^b^** **n (%)**	
	**Agree**	**Disagree**	**Don't know**	**Agree**	**Disagree**	**Don't know**
	
Emerging medical research is uncovering complexities of CFS	506 (40)	233 (19)	511 (41)	745 (50)	383 (25)	372 (25)
Enough information available to clinicians to diagnose CFS	308 (25)	604 (48)	338 (27)	448 (30)	876 (58)	176 (12)
CFS can impair a person's quality of life	1088 (87)	27 (2)	135 (11)	1353 (90)	79 (5)	68 (5)
I believe CFS is only in the patient's head	247 (20)	637 (51)	366 (29)	289 (19)	1077 (72)	134 (9)
If diagnosed and treated within first five years, CFS patients are more likely to get better	383 (31)	214 (17)	653 (52)	592 (40)	394 (26)	514 (34)
There are several treatment options available for people with CFS	543 (43)	300 (24)	407 (33)	738 (49)	463 (31)	299 (20)
A diagnosis of CFS can inhibit a patient's motivation to get better	685 (55)	208 (16)	357 (29)	849 (57)	458 (30)	193 (13)
CFS can impair quality of life for the patient's family or caregiver	1076 (86)	31 (3)	143 (11)	1341 (89)	82 (6)	77 (5)

^a ^N = 1250

^b ^N = 1500

We further examined how a history of making a CFS diagnosis influences attitudes and beliefs of CFS (Table 7). In 2006, 46% (n = 571) of DocStyles physicians reported having diagnosed CFS and 2007, 36% (n = 536) reported ever making a diagnosis of CFS (see Table 5). In both study years, physicians who had ever made a diagnosis of CFS had significantly different opinions as compared to those physicians who had not made a diagnosis. For example, in 2006, 36% of physicians who reported giving a diagnosis agreed that enough information was available to diagnose CFS compared to 15% who had not given a diagnosis. Physicians were less likely to think that CFS was in a patient's head if he/she had given a diagnosis (p < 0.01 in 2006; p < 0.01 in 2007).

**Table 7:** DocStyles Physicians History of CFS Diagnosis and CFS KABs.

2006 n (%) Have you ever given a diagnosis of CFS		Yes			No		
	Agree	Disagree	Don't know	Agree	Disagree	Don't Know	χ^2^
Emerging medical research is uncovering complexities of CFS	296 (52)	89 (16)	186 (32)	210 (31)	144 (21)	325 (48)	56.5***
Enough information available to clinicians to diagnose CFS	206 (36)	227 (39)	138 (24)	102 (15)	377 (56)	200 (30)	74.9***
CFS can impair a person's quality of life	513 (90)	7 (1)	51 (9)	575 (85)	20 (3)	84 (12)	8.6*
I believe CFS is only in the patient's head	75 (13)	375 (66)	121 (21)	172 (25)	262 (39)	245 (36)	91.5***
If diagnosed and treated within first five years, CFS patients are more likely to get better	220 (39)	91 (16)	260 (45)	163 (24)	123 (18)	393 (58)	31.3***
There are several treatment options available for people with CFS	313 (55)	137 (24)	121 (21)	230 (34)	163 (24)	286 (42)	73.0***
A diagnosis of CFS can inhibit a patient's motivation to get better	300 (53)	126 (22)	145 (25)	385 (57)	82 (12)	212 (31)	23.3***
CFS can impair quality of life for the patient's family or caregiver	512 (90)	10 (2)	49 (8)	564 (83)	21 (3)	94 (14)	11.3**

**2007 n (%) Have you ever given a diagnosis of CFS**		**Yes**			**No**		
	**Agree**	**Disagree**	**Don't** **know**	**Agree**	**Disagree**	**Don't** **know**	**χ^2^**

Emerging medical research is uncovering complexities of CFS	336 (63)	141 (26)	59 (11)	409 (42)	242 (25)	313 (33)	92.6***
Enough information available to clinicians to diagnose CFS	226 (42)	295 (55)	15 (3)	222 (23)	581 (60)	161 (17)	100.6***
CFS can impair a person's quality of life	503 (94)	28 (5)	5 (1)	850 (88)	51 (5)	63 (7)	25.1***
I believe CFS is only in the patient's head	62 (12)	546 (85)	18 (3)	227 (24)	621 (64)	116 (12)	75.1***
If diagnosed and treated with first five years, CFS patients are more likely to get better	278 (52)	155 (29)	103 (19)	314 (33)	239 (25)	411 (43)	89.9***
There are several treatment options available for people with CFS	349 (65)	160 (30)	27 (5)	389 (40)	303 (31)	272 (28)	136.0***
A diagnosis of CFS can inhibit a patient's motivation to get better	307 (57)	204 (28)	25 (5)	542 (56)	254 (26)	168 (17)	59.2***
CFS can impair quality of life for the patient's family or caregiver	504 (94)	26 (5)	6 (1)	837 (87)	56 (6)	71 (7)	28.8***

^a ^N = 1250

^b ^N = 1500

* p < 0.05; ** p < 0.01; *** p < 0.001

Physicians were asked to identify symptoms of CFS (Table 8). For each year, the majority of physicians correctly recognized symptoms associated with CFS and responded in the negative regarding symptoms not associated with CFS. The 4 most correctly identified symptoms (unexplained fatigue, unrefreshing sleep, impaired memory, and muscle or joint pain) were endorsed by over 80% of physicians.

**Table 8:** Percent of DocStyles Physicians Recognizing CFS symptoms.

Symptoms	Physicians	
	**2006** **n (%)**	**2007** **n (%)**
*Unexplained fatigue not improved by rest*	1179 (94)	1384 (92)
*Unrefreshing sleep*	1113 (89)	1291 (88)
*Impaired memory or concentration*	1025 (82)	1195 (80)
*Muscle or joint pain*	1049 (84)	1194 (80)
*Headaches*	848 (68)	937 (63)
*Tender lymph nodes*	533 (43)	660 (44)
*Sore throat*	472 (38)	519 (35)
Vomiting	64 (5)	90 (6)
Excessive thirst	66 (5)	87 (6)
Rash on trunk or extremities	64 (5)	77 (5)
Blood in urine	21 (2)	16 (1)

Italicized symptoms are CFS symptoms

2006 sample n = 1250

2007 sample n = 1500

During the 2006 DocStyles survey, 20% (n = 251) participants indicated they have read or heard something concerning in the past few months, as did 16% (n = 233) of 2007 DocStyles participants (Table 9). Those who said yes were further queried as to the source and the top 3 resources for both years were professional journals, the Internet, and continuing education programs.

**Table 9:** Source of DocStyles Physicians Reading or Hearing about CFS in Past Few Months.

Source	Physicians	
	**2006** **n (%)**	**2007** **n (%)**

Professional journals	153 (61)	(60)
Internet	96 (38)	(37)
Continuing education programs	67 (27)	(27)
Consumer magazines	50 (20)	(15)
Newspapers	40 (16)	(14)
Professional meeting	35 (14)	(12)
Television	40 (16)	(11)
Radio	25 (10)	(3)
Billboards	5 (2)	(1)
Photo Exhibit	1 (<1)	(<1)

2006 sample n = 251

2007 sample n = 233

## Discussion

We found parallel findings regarding healthcare providers' awareness, knowledge, and diagnosis concerning CFS in both the *Conference Healthcare Providers and DocStyles Physicians *samples. Over 96% of primary care physicians, pediatricians, and obstetrics/gynecologists in the *DocStyles *survey reported ever hearing about CFS. In both studies, physicians reported giving a diagnosis of CFS: 64% of conference attendee physicians, and an average of 41% of *DocStyles *physicians.

A history of prior CFS diagnosis by a healthcare provider or physician impacts the knowledge, attitudes, beliefs, and perceptions of how these professionals view CFS. Forty-one percent of C*onference Healthcare Providers *reported making a diagnosis of CFS and these respondents were more likely than those who had not made a diagnosis to have a better understanding of the complexities associated with CFS. Similarly, in 2006 and 2007, 46% and 36% of *DocStyles Physicians *reported giving a diagnosis of CFS and yet 20% of this sample agreed with the statement that "I believe that CFS is all in a patient's head." However, those physicians who had made a diagnosis were significantly less likely to agree with this statement, 66% in 2006 and 85% in 2007, compared to those who did not make a diagnosis, 39% and 64%. These findings are similar to those by Sheeres et al, who found that the more informed physicians are towards CFS, the better the knowledge, attitudes, and beliefs associated with CFS [13].

While additional research is needed to explore why a small percent of physicians who gave a diagnosis still agreed that "CFS is all in the patient's head," one hypothesis is that when physicians have a troublesome experience with a patient, it may skew their attitudes towards CFS and yet allow the diagnostic process to proceed. It must be noted that physicians may have difficulties with a CFS not because of the patient per say, but because the physician finds the diagnostic and management process difficult to navigate.

Physicians in the *Conference Healthcare Providers *sample had the highest mean score on knowledge compared to nurses, nurse practitioners and physician assistants, occupational therapists, and PhD/Masters. In terms of knowledge, more than 80% of physicians in the *DocStyles *survey correctly identified the 4 most common CFS symptoms (unexplained fatigue, unrefreshing sleep, impaired memory or concentration, and muscle or joint pain). U.S. physicians as a whole are clearly aware of CFS, knowledgeable about CFS symptoms, and around half have given a diagnosis.

Of all healthcare providers, nurses had the lowest mean knowledge compared to physicians, physician assistants, nurse practitioners, occupational therapists, and the PhD/Masters group. Providers who reported ever giving a diagnosis of CFS scored significantly higher on the knowledge factor compared to those who had not although this association was not statistically significant after the adjustment of physicians' degree and practice setting.

When examining beliefs towards CFS diagnosis and management, physicians, physician assistants, nurse practitioners, occupational therapists, and the PhD/Masters group all scored significantly higher than nurses, which indicates that nurses included in our sample did not have had an informed view of the diagnostic and management issue associated with CFS. Physicians and nurses had significantly higher scores on the attitude factor as compared to nurse practitioners, physician assistants, occupational therapists, and the PhD/Masters group, which indicates a more negative attitude towards CFS. However, all means in the attitudes factor fell between the 25^th ^and 50^th ^percentile indicating an overall low negative attitude to CFS among the sample. In the Perception score, physicians had the lowest mean score indicating a good perception of CFS in the population whereas nurses, nurse practitioners, and physician assistants had higher scores although not statistically significant.

Our finding that nurses in the *Conference Healthcare Providers *sample harbored slightly more negative attitudes and beliefs of CFS and were also less knowledge supports a recent study by Chew-Graham et al, which found that nurses in the United Kingdom had "limited knowledge and experience with clinical features of the illness, its etiology and appropriate management strategies" [[19], p 8]. Nurses in that study were aware of CFS, yet many reported receiving little training concerning the illness and this may account for the discrepancy in awareness versus education. In fact, many CFS education programs target healthcare providers who either diagnose CFS (e.g. physicians or nurse practitioners) or those who assist in management modalities (e.g. cognitive behavioral therapist or occupational therapists). While further research is warranted, CFS education programs aimed at nurses that provide information on the clinical aspects of the illness as well as overall management strategies may be beneficial.

In the United States less than 20% of persons with CFS receive a diagnosis and yet both the *Conference Healthcare Providers *and *DocStyles Physicians *respondents reported that an average of 41% of had diagnosed CFS. This finding suggests that persons with CFS may not be aware that assistance is available in the healthcare system in terms of CFS diagnosis and management. It is understandable that patients may delay care because of insurance issues or perceived barriers to healthcare access. Patients need encouragement to seek medical care for CFS symptoms and not concentrate on perceived attitudes and beliefs of providers. However, coupled with this approach, healthcare providers need further guidance on the clinical evaluation, diagnosis, and management strategies for CFS.

When physicians in the *DocStyles *survey responded to quality of life statements, over 86% of the sample agreed that CFS affects both the patient and the patient's family or caregiver quality of life. This confirms that U.S. physicians recognize the impact of CFS on patients and families. Contrary to existing beliefs, physicians in this sample showed compassion and understanding about CFS issues. In this same study physicians were asked about diagnostic information and treatment options for CFS. Fewer than 30% agreed that enough information was available to make a CFS diagnosis. In terms of treatment options, less than half of physicians agreed there were treatment options for CFS.

The disparity in agreement between acknowledgment that CFS affects quality of life, a high level of awareness, and the ability to recognize the symptoms of CFS versus the inability to recognize that diagnostic and treatment guides are available, point to the need for education programs that provide greater focus on the clinical evaluations and management options and less on raising CFS awareness. Data from both samples demonstrate that while a small percent of healthcare providers are skeptical of CFS and express negative attitudes, the majority of respondents recognize CFS as an illness and understand the impacts it has on society. While physicians in both samples showed high levels of CFS knowledge and appropriate attitude scores, they also demonstrated confusion in how to treat and manage CFS. Future CFS research and educational interventions should incorporate evidence-based behavioral theories with constructs of knowledge, attitudes, and beliefs in the development of programs.

Physicians in the *DocStyles *survey reported that professional journals, the Internet, and continuing education programs as the top three sources in which they had heard or read about CFS in the past few months. Indeed, the identification of appropriate dissemination and communication channels is extremely important to tailoring information to selected target audiences [20], and so should be taken more advantage of to have an even greater impact on healthcare professionals' awareness of CFS.

### Strengths and Limitations

A strength of the current research is the use of diverse research methods. It is common in social science research to combine convenience and probability sampling in order to reduce costs and yet strengthen the rigor of the study design. Other strengths of the study include sufficient sample sizes and the use of the factor analysis in the first sample to assess both reliability and construct validity of the survey.

This research has several limitations. In the *Conference Healthcare Providers *sample, the subjects were selected using convenience sampling, whereas in the second sample physicians were randomly selected proportional to the physician demographic database of the American Medical Association in terms of age, sex, and region. However, Singleton et al state that "while one should be mindful of [weaknesses] it would be a mistake to rule out nonprobability sampling," particularly "in the early stages of investigating a problem, when the objective is to become more informed about the problem itself." [[21], p 159].

A second limitation is the potential for selection bias in the *Conference Healthcare Providers *sample. While advertisements invited all healthcare providers at conferences to visit the CFS education booth and receive a free USB stick, there is the potential bias of persuasive efforts that those persons familiar with CFS or those who wanted a free USB stick, may have been more likely to visit the booth and participate in the survey. A third limitation is that physicians who participated in the *DocStyles *sample, a web-based survey, were more likely to be younger and more sophisticated with computers. The average age among this population was 44.6 years of age, which supports the view that the sample consisted of a younger group of physicians. Younger physicians may view CFS differently than older physicians as it is more likely that younger physicians have encountered CFS in medical school, residency programs, or continuing medical education. Physicians who use computers in their practice may differ from other practicing physicians by living or practicing in a more urban or metropolitan area as compared to those in rural areas. Finally, the questionnaire in the *DocStyles *sample was not assessed for reliability or validity.

## Conclusion

The findings begin to fill a gap in the evidence-base concerning knowledge, attitudes, beliefs, and perceptions of U.S. healthcare providers concerning CFS. However, further research concerning CFS KABs among healthcare providers and physicians is needed to assist in developing and delivering educational interventions. Importantly, participants in both samples expressed similar opinions in terms of knowledge, attitudes, and beliefs.

## Competing interests

The authors declare that they have no competing interests.

## Authors' contributions

DB drafted the manuscript, participated in study coordination, and helped to conceive the study. FF coordinated parts of the study design and helped to draft the manuscript. JML analyzed the data and contributed to drafting the manuscript. WR conceived of the study, participated in study coordination and helped to draft the manuscript. All authors read and approved the final manuscript.

## Pre-publication history

The pre-publication history for this paper can be accessed here:

http://www.biomedcentral.com/1471-2296/11/28/prepub

## Supplementary Material

Additional file 1 **Supplementary Table 1.** Bivariate Associations of CFS Knowledge, Attitudes, Beliefs, and Perception Domain Scores. Click here for file
