# Supplementary material for: U.S. healthcare providers' knowledge, attitudes, beliefs, and perceptions concerning Chronic Fatigue Syndrome
Source: BMC Fam Pract. 2010 Apr 21;11:28. doi: 10.1186/1471-2296-11-28 (PMC2875206; doi:10.1186/1471-2296-11-28)
Supplement: Additional file 1 — Supplementary Table 1. Bivariate Associations of CFS Knowledge, Attitudes, Beliefs, and Perception Domain Scores. [file 1471-2296-11-28-S1.doc]

**Additional File 1.**

**Supplementary Table 1. Bivariate Associations of CFS Knowledge, Attitudes, Beliefs, and Perception Domain Scores**

| **Bivariate associations between Attitude and subject characteristics** | | | | | | |
| --- | --- | --- | --- | --- | --- | --- |
|  |  | Mean | SD | β | SE(β) | p-value |
| Degree | |  |  |  |  | **<0.0001** |
|  | MD/DO | 8.1 | 3.4 | -0.02 | 0.41 | 0.9549 |
|  | **NP/PA** | 6.9 | 3.3 | -1.24 | 0.42 | **0.0034** |
|  | **OT** | 6.2 | 2.7 | -1.91 | 0.49 | **0.0001** |
|  | PhD/Masters | 6.7 | 3.6 | -1.41 | 0.62 | 0.0224 |
|  | Other | 6.5 | 2.7 | -1.62 | 0.66 | 0.0144 |
|  | RN | 8.1 | 3.6 | Reference |  |  |
| Practice Setting | |  |  |  |  | **0.0010** |
|  | Hospital | 7.8 | 3.5 | 0.78 | 0.37 | **0.0340** |
|  | Private Practice | 7.8 | 3.6 | 0.84 | 0.37 | **0.0224** |
|  | Group Practice | 7.3 | 3.1 | 0.31 | 0.39 | 0.4321 |
|  | Academic | 6.8 | 3.1 | -0.15 | 0.45 | 0.7365 |
|  | Other | 6.7 | 3.3 | -0.28 | 0.41 | 0.5035 |
|  | Community | 6.9 | 3.0 | Reference |  |  |
| Ever given a CFS diagnosis | |  |  |  |  |  |
|  | Yes | 7.6 | 3.5 | 0.20 | 0.20 | 0.3150 |
|  | Not Applicable | 6.8 | 3.0 | -0.61 | 0.39 | 0.1174 |
|  | No | 7.3 | 3.3 | Reference |  |  |
| **Bivariate associations between Belief and subject characteristics** | | | | | | |
|  |  | Mean | SD | β | SE(β) | p-value |
| Degree | |  |  |  |  | **<0.0001** |
|  | **MD/DO** | 10.5 | 2.6 | 1.37 | 0.33 | **<0.0001** |
|  | **NP/PA** | 10.3 | 2.8 | 1.23 | 0.34 | **0.0004** |
|  | **OT** | 9.9 | 2.7 | 0.79 | 0.40 | **0.0475** |
|  | **PhD/Masters** | 10.1 | 2.9 | 1.07 | 0.50 | **0.0344** |
|  | Other | 8.4 | 2.5 | -0.71 | 0.54 | 0.1868 |
|  | RN | 9.1 | 2.9 | Reference |  |  |
| Practice Setting | |  |  |  |  | 0.2189 |
|  | Hospital | 10.2 | 2.7 | 0.30 | 0.30 | 0.3132 |
|  | Private Practice | 10.4 | 2.8 | 0.49 | 0.30 | 0.0994 |
|  | Group Practice | 10.2 | 2.7 | 0.31 | 0.32 | 0.3263 |
|  | Academic | 9.7 | 2.9 | -0.24 | 0.39 | 0.5174 |
|  | Other | 10.3 | 2.7 | 0.35 | 0.33 | 0.2851 |
|  | Community | 10.0 | 3.0 | Reference |  |  |
| Ever given a CFS diagnosis | |  |  |  |  | 0.4937 |
|  | Yes | 10.2 | 2.7 | -0.07 | 0.16 | 0.6858 |
|  | Not Applicable | 10.3 | 2.7 | -0.37 | 0.31 | 0.2381 |
|  | No | 9.9 | 2.6 | Reference |  |  |
| **Bivariate associations between Knowledge and subject characteristics** | | | | | | |
|  |  | Mean | SD | β | SE(β) | p-value |
| Degree | |  |  |  |  | **<0.0001** |
|  | **MD/DO** | 10.3 | 2.4 | 1.08 | 0.31 | **0.0004** |
|  | **NP/PA** | 10.1 | 2.6 | 0.89 | 0.31 | **0.0049** |
|  | OT | 9.6 | 2.1 | 0.35 | 0.37 | 0.3475 |
|  | PhD/Masters | 9.6 | 2.3 | 0.38 | 0.46 | 0.4074 |
|  | Other | 8.6 | 1.8 | -0.65 | 0.50 | 0.1894 |
|  | RN | 9.2 | 2.9 | Reference |  |  |
| Practice Setting | |  |  |  |  | 0.4937 |
|  | Hospital | 10.1 | 2.6 | 0.14 | 0.27 | 0.5964 |
|  | Private Practice | 10.1 | 2.5 | 0.17 | 0.27 | 0.5296 |
|  | Group Practice | 10.1 | 2.6 | 0.17 | 0.29 | 0.4642 |
|  | Academic | 10.0 | 2.2 | 0.08 | 0.34 | 0.8132 |
|  | Other | 9.9 | 2.5 | -0.04 | 0.30 | 0.8916 |
|  | Community | 10.0 | 2.5 | Reference |  |  |
| Ever given a CFS diagnosis | |  |  |  |  | **<0.0001** |
|  | **Yes** | 10.4 | 2.5 | 0.64 | 0.15 | **<0.0001** |
|  | Not Applicable | 9.9 | 2.1 | 0.13 | 0.29 | 0.6572 |
|  | No | 9.8 | 2.5 | Reference |  |  |
| **Bivariate associations between Perception and subject characteristics** | | | | | | |
|  |  | Mean | SD | β | SE(β) | p-value |
| Degree | |  |  |  |  | **0.0065** |
|  | MD/DO | 7.3 | 2.4 | 0.14 | 0.32 | 0.6159 |
|  | NP/PA | 8.3 | 2.7 | 0.16 | 0.33 | 0.6250 |
|  | OT | 7.6 | 2.6 | -0.55 | 0.38 | 0.1444 |
|  | PhD/Masters | 7.4 | 2.6 | -0.72 | 0.48 | 0.1333 |
|  | Other | 7.3 | 2.0 | -0.79 | 0.51 | 0.1258 |
|  | RN | 8.1 | 2.6 | Reference |  |  |
| Practice Setting | |  |  |  |  | **0.0405** |
|  | Hospital | 8.3 | 2.5 | -0.13 | 0.27 | 0.6410 |
|  | Private Practice | 8.3 | 2.5 | -0.01 | 0.27 | 0.8068 |
|  | Group Practice | 7.9 | 2.3 | -0.43 | 0.30 | 0.1448 |
|  | Academic | 8.0 | 2.6 | -0.40 | 0.35 | 0.2482 |
|  | Other | 7.6 | 3.0 | -0.77 | 0.31 | **0.0135** |
|  | Community | 8.3 | 2.7 | Reference |  |  |
| Ever given a CFS diagnosis | |  |  |  |  | 0.0650 |
|  | Yes | 8.2 | 2.5 | 0.02 | 0.15 | 0.8933 |
|  | Not Applicable | 8.2 | 2.6 | -0.65 | 0.29 | **0.0250** |
|  | No | 7.5 | 2.6 | Reference |  |  |

β indicates the coefficient in the linear model and SE indicates the standard error of β estimate.
